# Synthesis of lignin-derived nitrogen-doped carbon as a novel catalyst for 4-NP reduction evaluation

**DOI:** 10.1038/s41598-020-76039-9

**Published:** 2020-11-18

**Authors:** Yun Liu, Huanghui Xu, Hongfei Yu, Haihua Yang, Tao Chen

**Affiliations:** 1 College of Life Science and Technology, Beijing University of Chemical Technology, Beijing, 100029 China; 2 School of Nuclear Technology and Chemistry and Biology, Hubei University of Science and Technology, Xianning, 437100 China; 3 Hubei Key Laboratory of Radiation Chemistry and Functional Materials, Hubei University of Science and Technology, Xianning, 437100 China; 4 Hubei Engineering Research Center for Fragrant Plants, Hubei University of Science and Technology, Xianning, 437100 China

**Keywords:** Chemistry, Materials science

## Abstract

In this study, nitrogen-doped carbon (NC) was fabricated using lignin as carbon source and g-C_3_N_4_ as sacrificial template and nitrogen source. The structural properties of as-prepared NC were characterized by TEM, XRD, FT-IR, Raman, XPS and BET techniques. Attractively, NC has proved efficient for reducing 4-nitrophenol (4-NP) to 4-aminophenol (4-AP) using NaBH_4_ as hydrogen donor with high apparent rate constant (*k*_app_ = 4.77 min^−1^) and specific mass activity (s = 361 mol kgcat^−1^ h^−1^), which values are superior to the previously reported catalysts in the literature. Density functional theory (DFT) calculations demonstrate that four kinds of N dopants can change the electronic structure of the adjacent carbon atoms and contribute to their catalytic properties dependant on N species, however, graphitic N species has much greater contribution to 4-NP adsorption and catalytic reduction. Furthermore, The preliminary mechanism of this transfer hydrogenation reaction over as-prepared NC is proposed on the basis of XPS and DFT data. Astoundingly, NC has excellent stability and reusability of six consecutive runs without loss of catalytic activity. These findings open up a vista to engineer lignin-derived NC as metal-free catalyst for hydrogenation reaction.

## Introduction

Lignin, the second-most abundant renewable aromatic biopolymer in nature, accounts for 15–30 wt% of lignocellulosic biomass. It has been taking hold of the scientific community agenda in the biomass biorefinery^1–3^. Depolymerization through oxidation and/or reduction strategies into monomeric, aromatic compounds is the current prevalent method of lignin valorisation^4–6^. In 2014, Rahimi et al. described a method for the depolymerization of oxidized lignin under mild conditions in aqueous formic acid that resulted in more than 60 wt% yield of low-molecular-mass aromatics^7^. In 2018, Wu et al. reported an electron–hole coupled photoredox mechanism to cleavage β-O-4 bond in lignin with the yield of about 27 wt% of aromatic monomers from birch woodmeal^8^. Besides efforts in the depolymerization of lignin towards low-molecular-mass aromatics, attempts to convert this polymer into lignin-derived carbon-based catalysts have been recently reported^9–11^. For instance, in 2019, Zhou et al. developed the assembly of lignin–metal complexes for producing bimetallic nanoparticles catalyst and/or metal single-atom catalysts supported on nitrogen-doped carbon using lignin as covalent ligand, which opens new avenue towards lignin-derived functional catalytic materials^10,11^.

In comparison with metallic catalysts, metal-free catalysts like carbon materials have many outstanding merits of low-cost, bio-degradation, environmental friendly and readily available aspects, which can avoid heavy metals environmental pollution and depletion of rare metal resources^12–15^. Heteroatomic dopants, e.g. S-, B-, P- and N-doped carbon materials can change electronic density structure of the adjacent carbon atoms and promote their catalytic activities, they have been successfully applied in electrochemical reaction^16^, supercapacitor electrodes^17^, catalytic oxidation^18^, and reduction reaction^19^. For instance, Lin et al. described a boron-doped carbon material that showed excellent chemoselective reduction ability of nitroarenes with good conversions (99%) for substrates and selectivities (88%) for desired products^20^. Duan et al. synthesized a N,P-codoped carbon exhibiting excellent activity and exclusive selectivity for catalytic transfer hydrogenation of nitroarenes^21^. More recently, N-doped reduced graphene oxide (NG) has been found to be an effective metal-free catalyst for 4-NP reduction, a key transformation in fine chemical synthesis^19,21–23^. In the last decades, extensive investigations on this reaction have been conducted over both metallic catalysts^22,24–26^ and metal-free catalysts^12,19,20,23,27^.

In the present work, a lignin-derived N-doped carbon (NC) was synthsized through evaporation-induced deposition approach followed by carbonization treatment. It could be used as efficient metal-free catalyst for the conversion of 4-nitrophenol (4-NP) to 4-aminophenol (4-AP) with NaBH_4_ as hydrogen donor. To our best knowledge, no studies have been yet reported to synthesize NC using lignin as carbon source and g-C_3_N_4_ as soft template and N source, which has a high N content (57.1%) and complete decomposition at temperature above 710 °C^28^. In our work, the main objectives are to investigate the effect of lignin/g-C_3_N_4_ mass weight ratio and annealing temperature on the structure and activity of the desired NC catalysts. Furthermore, density function theory (DFT) is employed to address two critical issues for 4-NP reduction over the as-prepared NC. One is to state the preferred binding sites related to nitro group and/or the O atom of hydroxyl substitutes. The other is to examine the contributions of four kinds of nitrogen dopants to catalytic activity. In addition, the reaction kinetics and stability of the as-prepared NC are evulated for 4-NP reduction. According to experimental data and DFT calculations, a plausible catalytic mechanism of 4-NP over this desired NC catalyst is proposed. These findings in our work will pave a new avenue about lignin valorisation towards nitrogen-doped carbon-based catalyst for hydrogenation reaction.

**Scheme 1:**
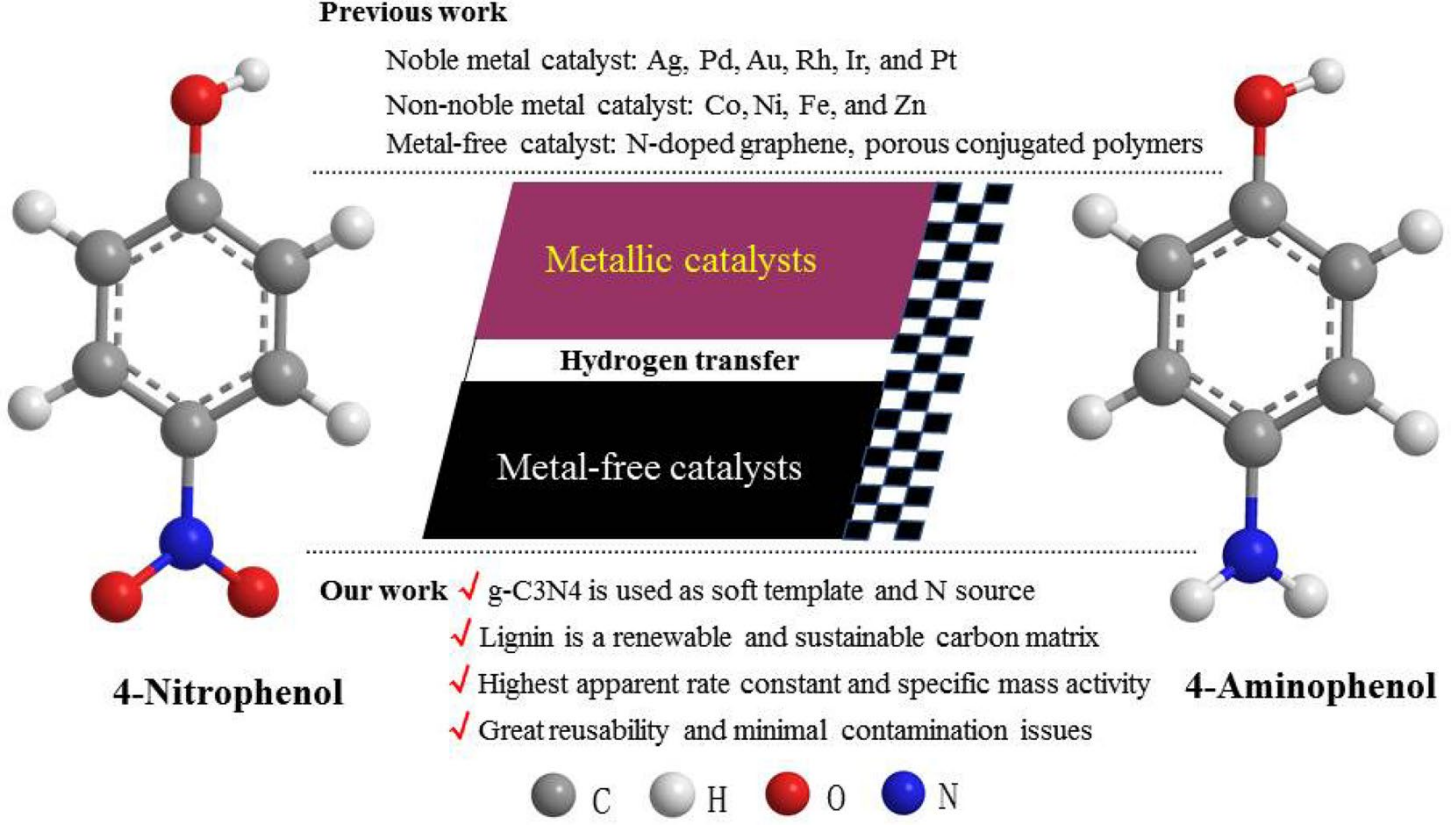
Overviews of 4-nitrophenol (4-NP) reduction to 4-aminophenol (4-AP) through hydrogenation reaction by different catalysts in previous and our works.

As shown in Scheme 1, compared with the earlier reported articles, several outstanding merits will be found in this present work, (1) g-C_3_N_4_ with high surface specific area (S_BET_ = 1200 m^2^/g) and high N content (57.1%) is used as sacrificial template (decomposition at 710 °C) and N source; (2) low-cost abundant lignin is employed as a renewable carbon matrix; (3) the as-prepared NC catalyst with high apparent rate and mass specific activities for 4-NP reduction outperforms most of the previous reported catalysts in the literature; (4) the as-prepared NC catalyst shows excellent re-usability and causes no environmental contamination issues through catalyst recycling.

## Materials and methods

### Lignin isolated from *Eucalyptus* biomass using aqueous formic acid (FA) fractionation

Lignin was isolated from eucalyptus wood using formic acid aqueous solution according to the modified procedures detailed in the literature^29^, and the FA fractionation procedure was shown in Supplementary Fig. S1. Briefly, 445 mL FA with the concentration of 70% was filled in 2 L round-bottom glass flask, which was loaded with 44 g *Eucalyptus* powder. The solid mass weight (g) to liquid volume (mL) ratio was set at approx. 1:100. The mixture was stirring at 1300 rpm and 130 °C for 3 h. After the desired reaction time, the mixture was filtered. The black liquor filtrate was collected and evaporated in vacuum condition to recover FA. After FA recovery, lignin was precipitated at the bottom of the flask. The precipitated lignin was washed with 300 mL distilled water and then filtered to separate lignin from aqueous phase. After dried, approx. 9 g of lignin pellets were obtained. According to the method detailed in our previous work^30^, the compositions of the obtained lignin were calculated to be 90.31% of lignin with small amount of cellulose (0.8%), hemicellulose (0.24%) and others (2.43%). The morphology of lignin show sphere appearance and the particle size of lignin nanoparticle is approx. 83 nm (Supplementary Fig. S1).

### g-C_3_N_4_ soft temple preparation

g-C_3_N_4_ was prepared by high-thermal decomposition polymerization of urea^31^. Typically, 28 g of urea was transferred to 50 mL lidded porcelain crucible, and then heated in a muffle furnace from room temperature to 550 °C at the heating rate of 2 °C min^−1^ and kept at 550 °C for 4 h. After cooling to room temperature, about 1.81 g of g-C_3_N_4_ was obtained in our work. The surface specific area (S_BET_) of the obtained g-C_3_N_4_ sample was measured to be 1200 m^2^/g) and its total N content was 57.1%.

### Lignin-derived nitrogen-doped carbon (NC) catalyst synthesis through evaporation-induced deposition approach followed with carbonization treatment

The synthesis procedure of evaporation-induced deposition approach was conducted to prepare lignin-derived nitrogen-doped carbon (NC) catalyst. Typically, the amount of g-C_3_N_4_ was dispersed in 100 mL of 70% FA aqueous solution, in which 1 g lignin with the final concentration of 1 wt% was dissolved under ultrasonic condition for 30 min. The mass weight ratio of g-C_3_N_4_ to lignin was set at 2, 6, 10, 14 and 18. The FA in the resulting suspension was evaporated at 45 °C under vacuum condition and lignin nanoparticles were homogeneously deposited onto the surface of g-C_3_N_4_. Afterwards, five samples (g-C_3_N_4_@lignin) dependant on the mass ratio of g-C_3_N_4_/lignin were collected and dried in an oven at 80 °C overnight. Five g-C_3_N_4_@lignin products were milled in agate mortar for approx. 45 min. About 3 g of powder was tiled in porcelain boat and calcined at 1073 K, 1173 K, 1273 K and 1373 K, respectively for 2 h in an argon atmosphere to yield a score of N_x_C-T catalysts (x represents the N content (x = 2, 6, 10, 14 and 18), T = 1073 K, 1173 K, 1273 K and 1373 K, respectively). The experimental design of N_x_C-T catalysts syntheses was shown in Supplementary Fig. S2. As a control, sole lignin without g-C_3_N_4_ template was annealed at 1373 K for 2 h in an argon atmosphere, it was labeled as LC-1373.

### Structural properties characterization of N_x_C-T catalysts

The morphology of transmission electronic microscope (TEM) image was collected on a Hitachi H-800 spectroscope (Japan) operating at 10 kV. Fourier Transform infrared spectroscopy (FT-IR) profiles were recorded on a Bruker Vertex 70 (German) from 400 to 4000 cm^−1^ at a resolution of 2 cm^−1^, equipped with a temperature- controlled attenuated total reflectance (ATR) device with a ZnSe crystal (Pike Technology). X-ray photoelectron spectroscopy (XPS) was performed on a ThermoFisher Scientific ESCALAB 250XI (USA) using monochromated Al Kalph source (150 W, 500 μm). The pass energy was 50 eV for survey, and 30 eV for high resolution scans. All binding energies were reference to the C1s peak at 284.4 eV. X-ray diffraction (XRD) patterns were recorded on a Bruker D2-phaser diffractometer (German) at 40 mA and 40 kV using Cu Kα radiation (λ = 1.54, 6.88°/min from 5 to 90°). Raman spectra were recorded on a Renishaw inVia Micro-Raman Spectroscopy System (England) equipped with charge-coupled device detector at 633 nm. Specific surface areas and pore size distributions were determined by Brunauer Emmett-Teller (BET) method on Micromeritics ASAP 2460 apparatus (USA) from nitrogen sorption isotherms collected at 77 K. The samples were degassed at 250 °C for 12 h prior to measurement.

### Catalytic performance of 4-NP reduction over NxC-T catalysts

140.5 mg of 4-NP was dissolved in 100 mL deionized water using as stock solution and kept in a brown bottle at 4 °C for use. Then, 5 mg of NC catalysts were homogeneously dispersed in 200 mL of deionized water under ultrasonic condition for 5 min and immersed into water bath at 25 °C under stirring condition for 10 min. Subsequently, 1.6 mL of 4-NP and 6.4 mmol of NaBH_4_ were added into the solution. After a certain regular time interval, an aliquot of 4 mL was taken from the reaction mixture. The NC catalyst was separated by filtering via 0.22 μm membrane, and the obtained transparent solution was used for absorption spectral analysis by UV756 UV–visible spectrophotometer (Shanghai, China).

Reaction kinetics of 4-NP reduction over NC catalyst was investigated using NaBH_4_ as hydrogen donor in water. Pseudo-first-order kinetics model was applied in our work because NaBH_4_ was greatly excessive and its concentration was regarded as being constant in the reaction^32^. Therefore, the apparent rate constant (*k*_*app*_) was calculated by Eq. (1):

1 $$ { - }k_{app} t = \ln \frac{{C_{t} }}{{C_{0} }} $$

where *C*_*0*_ and *C*_*t*_ was the concentration of 4-NP at initial and assigned reaction time (t), respectively. The value of *k*_*app*_ could be calculated from the slope of the fitting line $\ln \frac{{C_{t} }}{{C_{0} }} \to t$.

To assess the catalytic activity of the as-synthesized NC catalyst in our work, specific mass activity was evaluated according to Eq. (2):

2 $$ Specific\;mass\;activity = \frac{N}{{M_{cat} T}} $$

where N means the mole fraction of 4-NP at assigned reaction time, mol; M_cat_ is the mass weight of catalyst, kg; T represents the reaction time, h.

To investigate the effect of nitrogen dopant on the activity of lignin-derived NC catalysts, several counterparts including LC-1373, graphite powder, g-C_3_N_4_, graphene oxide (GO), and N_14_C-1373 were compared for the conversion of 4-NP to 4-AP. The catalysts LC-1373, g-C_3_N_4_ and N_14_C-1373 were synthesized in our lab, while graphite powder and GO were bought from Beijing local reagent company.

The recycling experiments of as-prepared NC catalyst were also examined for six runs. For each trial, the used catalyst was separated and washed with deionized water three times before next batch. Each reaction trial was run in triplicates and the average value was used as the final result in this work. For comparison, commercial Pb/C catalyst was employed in the 4-NP catalytic test under the same conditions to assess metallic catalytic activity and metal leaching.

### Density functional theory (DFT) calculation

To address the preferred binding sites of 4-NP over NC catalyst and nitrogen dopants contribution of NC catalyst to the catalytic performance, DFT simulations were performed by Vienna ab-initio simulation package (VASP) with the projector augmented wave pseudo-potentials (PAW) to describe the interaction between atomic cores and valence electrons^33^. The Perdew–Burke–Ernzerhof (PBE) function within the generalized gradient approximation (GGA) was used to implement DFT calculations. The N-doped graphene cluster models (20 Å × 20 Å × 12 Å) were employed to simulate the surface properties. The reasonable vacuum layers were set around 12 Å in the z-directions for avoiding interaction between planes. A cutoff energy of 400 eV was provided, and a 2 × 2 × 1 Monkhorst Pack k-point sampling was chosen for the well converged energy values. The optimum geometry structure of NC catalyst was pursued until the force on each atom fell below the convergence criterion of 0.02 eV/Å, and energies were converged within 10^–5^ eV^34,35^.

## Results and discussion

### Synthesis of lignin-derived NC catalyst and its structural characterization

**Figure 1:**
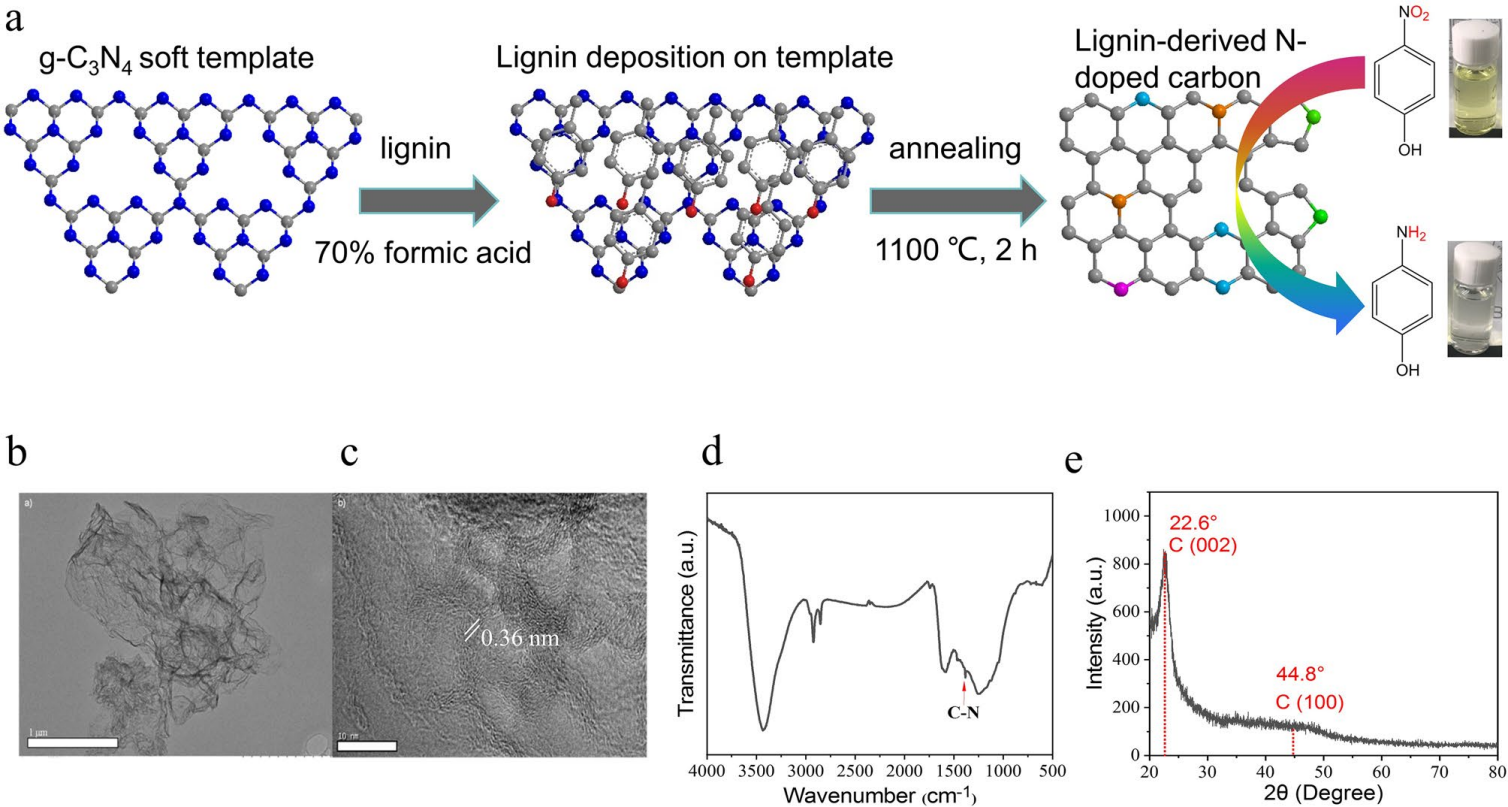
Lignin-derived NC synthesis and its structural properties. (**a**) Schematic overview for the synthesis procedure of lignin-derived NC at 1100 °C for 2 h. Lignin was dissovled in FA solution. After FA evaporation, lignin with sphere appearance and the particle size of approx. 83 nm was deposited on the surface of g-C_3_N_4_ template. (**b**) TEM image of N_14_C-1373. (**c**) HR-TEM image of N_14_C-1373. (**d**) FT-IR profile of N_14_C-1373. (**e**) XRD pattern of N_14_C-1373.

The synthesis procedure of lignin-derived NC was shown in Fig. 1a using lignin as carbon source and g-C_3_N_4_ as both nitrogen source and sacrificial template. The synthesis procedure was mainly divided into two steps, lignin deposition on template surface and calcination. In brief, g-C_3_N_4_ template was homogeneously dispersed in 70% FA solution, in which 1% lignin was dissolved. The mass weight ratio of g-C_3_N_4_ to lignin was set at 2, 6, 10, 14 and 18. After FA evaporation, lignin nanoparticles were deposited on the surface of template. Subsequently, the lignin/g-C_3_N_4_ was annealled at the assigned temperature for 2 h. Then 20 kinds of N_x_C-T catalysts were obtained in dependence of annealing temperature and N content (Supplementary Fig. S2; Supplementary Table S1). In our work, lignin was chosen as carbon source due to its abundance in carbon content (~ 60 to 70 wt%), sustainability and low-cost because lignin has been typically viewed as byproduct of biomass refinery^36^. While g-C_3_N_4_ was chosen as both template and N source due to its nitrogen-rich (57.1 at%), 3D pore structure and complete decomposition at above 700 °C^31^. To the bset of our knowledge, few articles have yet been available utilizing lignin as carbon source and g-C_3_N_4_ as both template and nitrogen source to fabricate NC catalyst. To our delight, the as-synthesized NC showed high catalytic activity for 4-NP reduction and outperformed most of the previously reported catalysts in the literature^19–27^.

Using N_14_C-1373 as an example, the morphology of the resultant NC was characterized by TEM. As shown in Fig. 1b, the NC sample is wrinkle veil-like thin nanosheet. It is the consequence of thermal reduction and exfoliation^19^. The high-resolution TEM image (Fig. 1c) further confirms that the nanosheet consists of 3–10 layers. The average distance of interlayer is 0.36 nm, similar to the interlayer distance of graphite (~ 0.34 nm). It is indicating that lignin is graphitizable at high annealing temperature of 1373 K. This phenomenon was demonstrated in our previous work, in which it was found that lignin started to graphitize at 600 °C and graphitizable carbon could be obtained at 1000 °C^10,11^. The FT-IR peak in Fig. 1d at approx. 1380 nm^−1^ is ascribed to C-N stretch, showing N atom is successfully doped into graphitizable carbon^27^, although the peak at 1380 cm^−1^ is weak. Further insight into the micro-structure of NC from XRD pattern (Fig. 1e), two peaks at 22.6° and 44.8° are observed, which is ascribed to (002) and (100) planes of graphite-like structure^12,37^, respectively. It indicates that the well-ordered graphene with 0.34 nm space is obtained after annealing process^19^.

**Figure 2:**
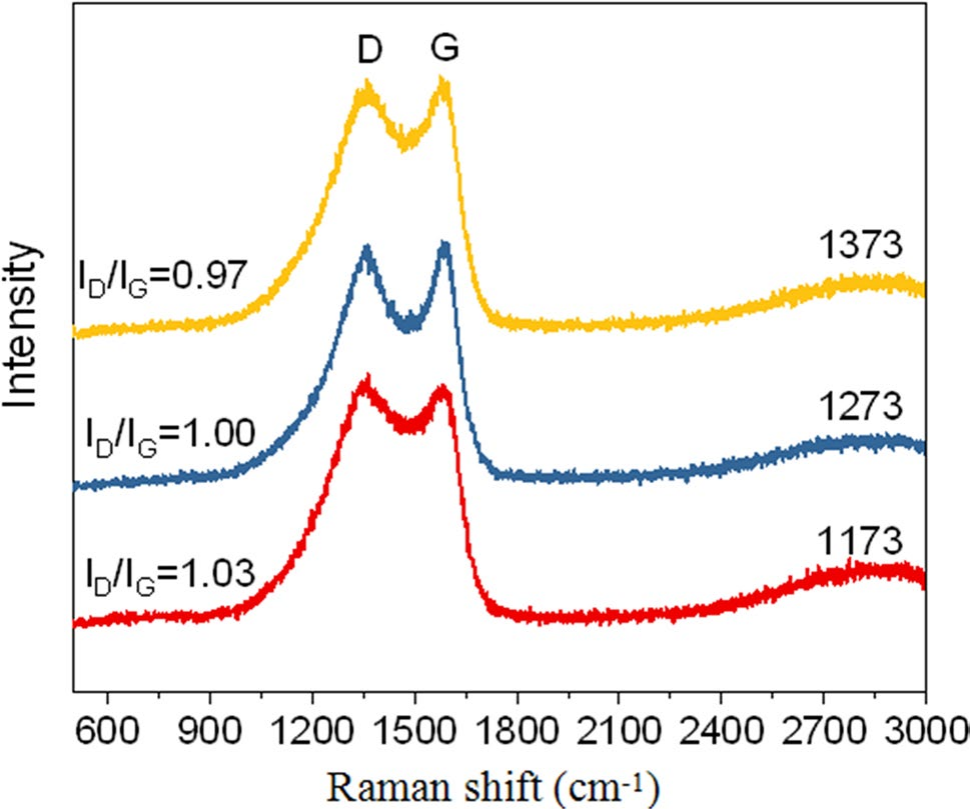
Raman spectra of N_14_C at different annealing temperatures of 1173, 1273 and 1373 K. The I_D_/I_G_ values represent the graphitizable degree of carbon material. The lower of the I_D_/I_G_ value, the higher of graphitizable degree of carbon material.

The influence of annealing temperature (1173 K, 1273 K and 1373 K) on the graphitizable degree of N_14_C was characterized by Raman spectra and shown in Fig. 2. As seen in Raman spectra profiles, peak at 1344 cm^−1^ assigned to disordered sp^3^ carbon (D band) and peak at 1599 cm^−1^ ascribed to graphitic sp^2^ carbon (G band) are observed. The I_D_/I_G_ values of N_14_C-1173, N_14_C-1273, N_14_C-1373 are 1.03, 1.00, and 0.97, respectively. It indicates that graphitizable degree of N_14_C increases with the increasing of the calcination temperature. The phenomenon is good consistence with the result reported in our previous work^10,11^. However, it is different from the phenomenon described by Yang et al., who pointed out that I_D_/I_G_ value of nitrogen-doped graphene (NG) decreased as the increasing temperature due to NG partially reduced at high temperature^19^.

**Figure 3:**
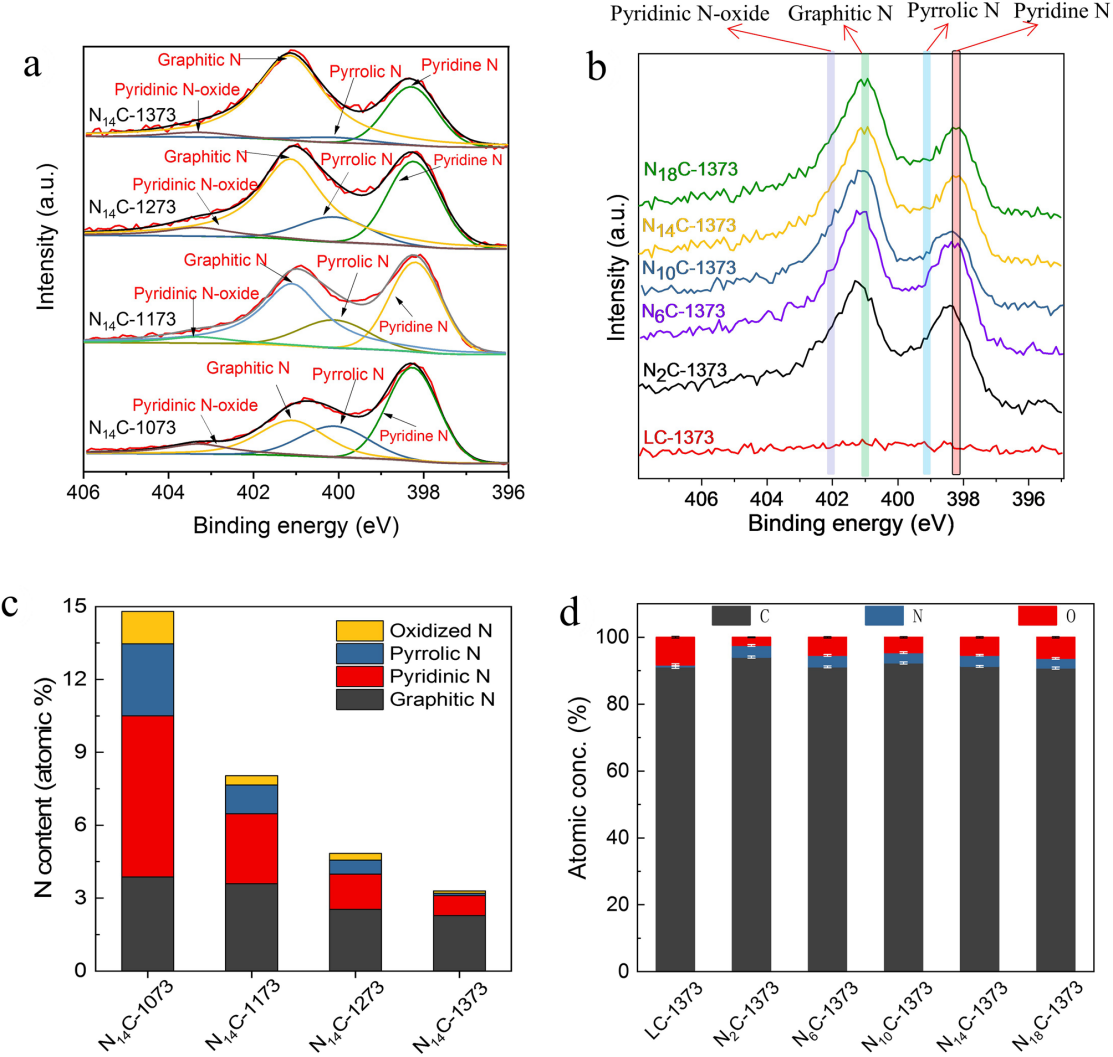
XPS spectra of NC at different annealing temperature and g-C_3_N_4_/lignin ratio. (**a**) N 1 s scans of NC at different temperature. (**b**) XPS spectra of NC at different g-C_3_N_4_/lignin ratio. (**c**) Nitrogen content of each type of N species at different temperature. (**d**) Element content of NC samples at different g-C_3_N_4_/lignin ratio.

To address the effect of g-C_3_N_4_/lignin ratio and annealing temperature on the elemental composition and N species of NC catalysts, XPS measurement was conducted and the results are shown in Fig. 3. The relative atom ratios of C, N and O element of NC samples are summarized in Supplementary Table S2.

Figure 3a shows the effect of annealing temperature on the types of N species of NC. The high resolution N1s XPS spectra can be deconvolved into four types of N species, namely, pyridinic N at 398.6 eV, pyrrolic N at 399.2 eV, graphite N at 400.5 eV and pyridinic N oxide at 402.6 eV, respectively^19,27^. The atomic concentration of total N in each sample is ranging from 3.3 at% to 14.8 at% (Fig. 3c). With an increase annealing temperature from 1073 to 1373 K, the content of total N in each sample decreases (Supplementary Table S2). It is probably ascribed to low thermal stability of nitrogen species at high temperature^19^. However, the graphite N species content in N_14_C-1073, N_14_C-1173, N_14_C-1273, and N_14_C-1373 is calculated to be 26 at%, 45 at%, 52 at% and 69 at%, respectively. Therefore, the ratios of the graphite N to the total N content increase with the enhancement of annealing temperature. The reasonable explanation is the fact that the graphite N species is thermo-stable and the total N content decreases due to other N species directly removing from the graphene sheet^19^. The variance of graphite N species in dependence of temperature is good consistence with the tendency of I_D_/I_G_ calculated from Raman spectra (Fig. 2).

Figure 3b shows the XPS spectra of NC in dependence of g-C_3_N_4_/lignin ratio at the same annealing temperature of 1373 K. It is worthly noticed that the amount of g-C_3_N_4_ has little influence on the N content (Fig. 3d). It is probably due to decomposition of g-C_3_N_4_ at high temperature, resulting in limited N-doped sites in graphitization carbon surface. This phenomenon is also confirmed by FT-IR detection in Fig. 1d. The XPS result (Supplementary Table S2) related to different g-C_3_N_4_/lignin ratio indicates a 27–31:1 ratio between C and N content.

**Figure 4:**
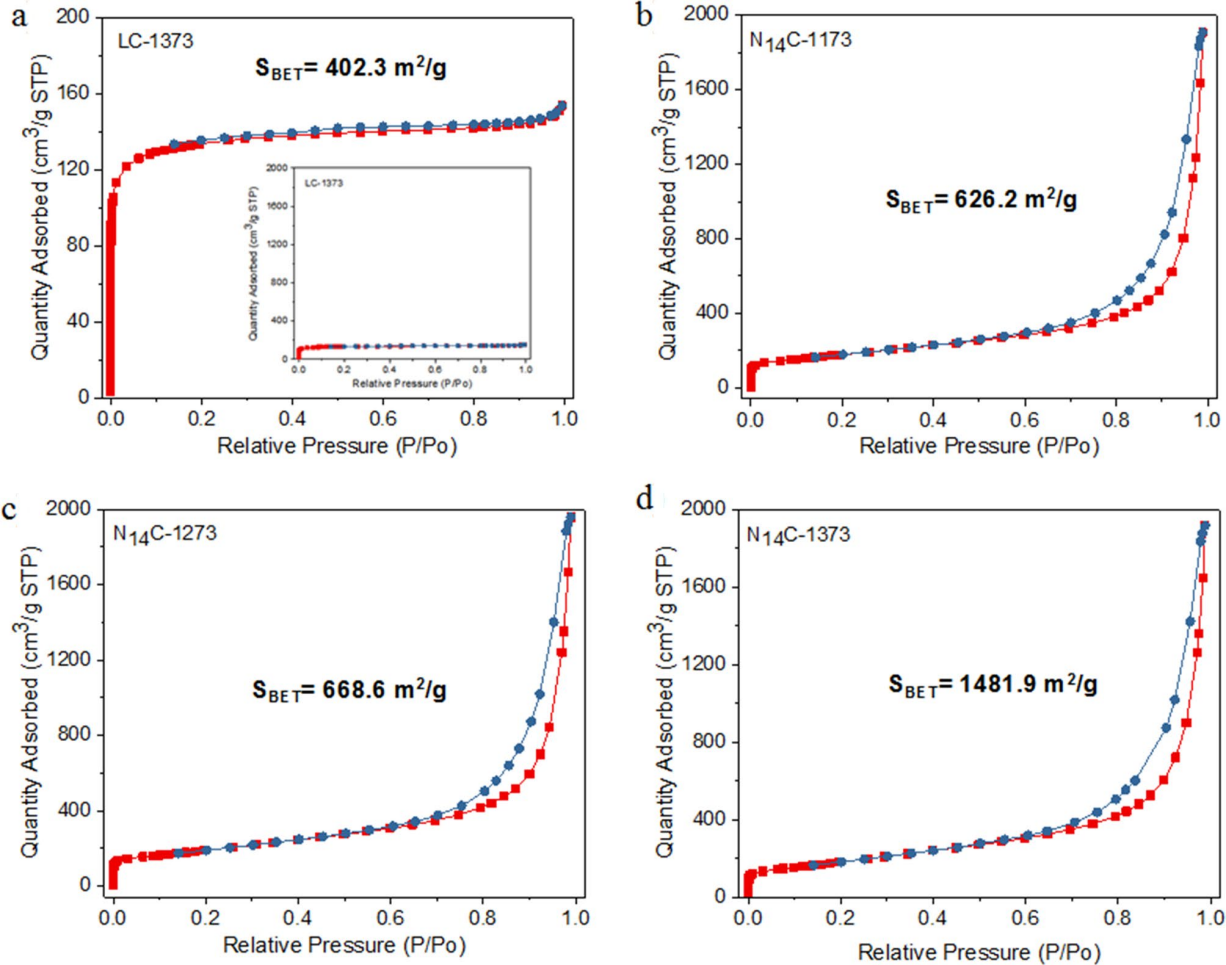
Effect of annealing temperature on BET surface area measurement of LC-1373 (**a**), N_14_C-1173 (**b**), N_14_C-1273 (**c**) and N_14_C-1373 (**d**).

Annealing temperature has significant effect on the specific surface area and pore diameter size of NC catalysts, which was determined by BET measurement using N_14_C-T as an example (Fig. 4). In absence of g-C_3_N_4_ template, the N_2_ adsorption isotherms for LC-1373 shows a type I curve (Fig. 4a). On the contrary, using g-C_3_N_4_ as soft template, the N_2_ adsorption isotherms for NC samples show a type IV curve with a hysterias loop (Fig. 4b–d), indicating both micro- and mesoporosity^38^. In comparison with LC-1373 (without g-C_3_N_4_ template, S_BET_ = 402.3 m^2^/g), the BET specific surface areas (S_BET_) determined for N_14_C-1173, N_14_C-1273 and N_14_C-1373 are 626.2 m^2^/g, 668.6 m^2^/g and 1481.9 m^2^/g, respectively. Their corresponding pore sizes were 19.5, 18.1, and 17.1 nm, respectively. It is evident that g-C_3_N_4_ serving as template is highly beneficial to achieving high S_BET_ of NC and additional mesoporosity into the samples^39^. In combination with the data in Raman spectra (Fig. 2), a reasonable conclusion has been drawn that the formation of ordered graphite-like layers is contribution to achieving high S_BET_ of the NC. For instance, N_14_C-1373 has the lowest value of I_D_/I_G_ (= 0.97) relative to N_14_C-1173 (I_D_/I_G_ = 1.03) and N_14_C-1273 (I_D_/I_G_ = 1.00), it shows the maximal S_BET_ (1481.9 m^2^/g). The structural change of carbon material occurring at high annealing temperature would affected its specific surface area and catalytic performance^14^.

### Catalytic performance of lignin-derived NC for 4-NP reduction

**Figure 5:**
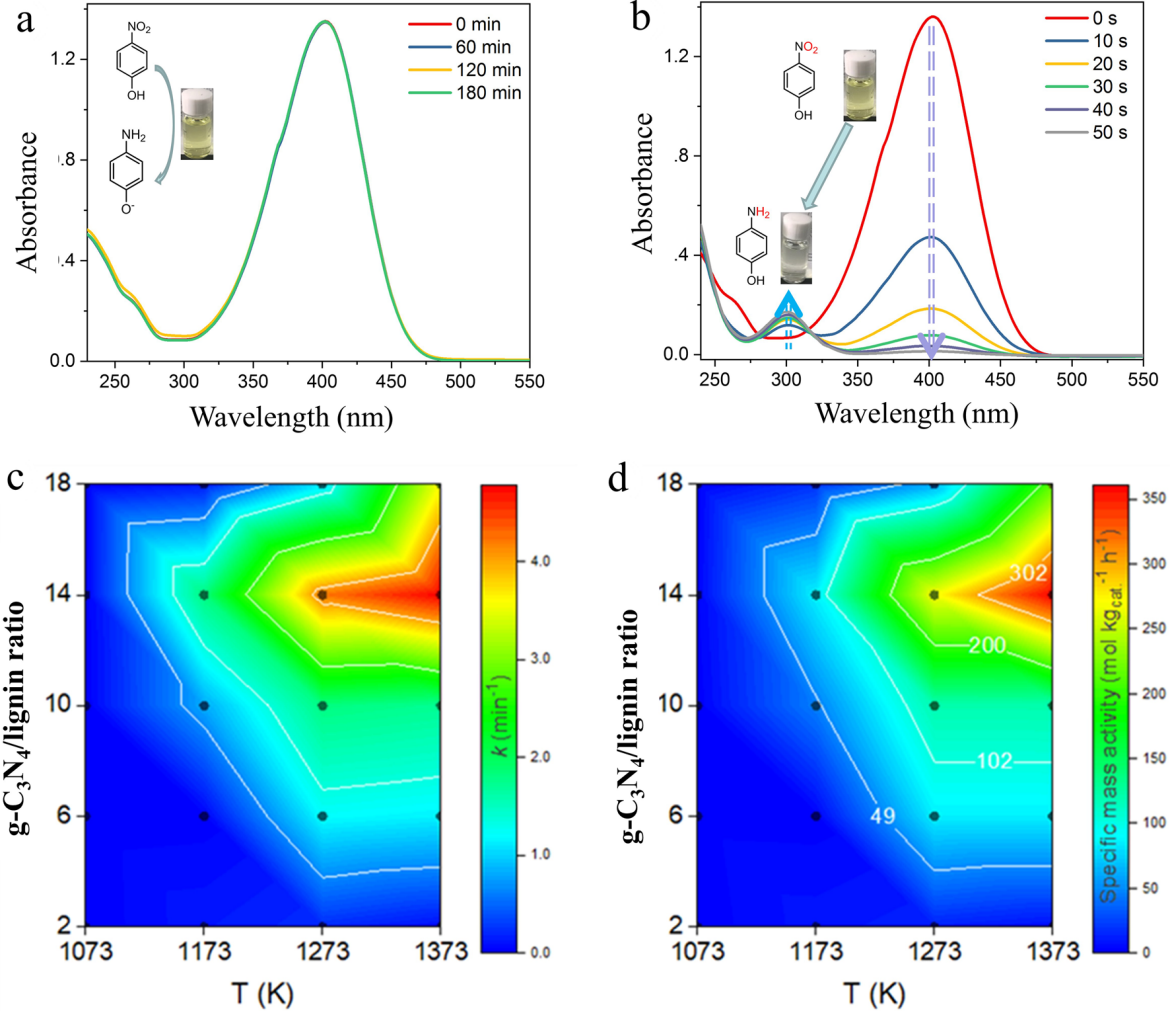
Catalytic performance of 4-NP reduction over lignin-derived NC catalyst. (**a**) Time-dependent UV–Vis spectra for the reduction of 4-NP with NaBH_4_ in the absence of catalyst; (**b**) time-dependent UV–Vis spectra for the reduction of 4-NP by NaBH_4_ in the presence of N_14_C-1373. (**c**) Effect of annealing temperature and g-C_3_N_4_/lignin ratio on *k*_*app*_ value. (**d**) Effect of annealing temperature and g-C_3_N_4_/lignin ratio on specific mass activity.

After elucidation of structural characterization, the catalytic performance of N_14_C-1373 was evaluated by 4-NP reduction to 4-AP with an excess NaBH_4_ in water. Interestingly, NaBH_4_ can not reduce 4-NP to 4-AP even after 3 h because of no observation of absorbance change determined by UV–Vis spectroscopy (Fig. 5a). However, the 4-NP solution color changes from light yellow to bright yellow with an absorbance at 400 nm after immediate addition of freshly prepared NaBH_4_ solution. It is ascribed to the formation of 4-nitrophenolate ions in alkaline condition^40^. Moreover, a lot of bubble are observed to release from 4-NP solution. It is the consequence of hydrogen gas generated by hydrolysis reaction of NaBH_4_^41^. Gratefully, when adding N_14_C-1373 catalyst, the color of the reaction mixture is immediately changed from yellow to complete colorless within 50 s, indicating 4-NP is reduced to produce 4-AP (Fig. 5b). UV–vis spectroscopy demonstrates that the absorption band of 4-nitrophenolate ion is at 400 nm, while the signal of 4-aminophenolate ion is at ~ 300 nm^19–21^. In Fig. 5b, two isosbestic points are also found at ~ 274 and 315 nm, a symbol of the complete conversion of 4-NP to 4-AP without the generation of intermediate byproducts, which has been considerably confirmed in the literature^23,39^. It is worthily noting that 4-NP reduction initiates immediately by adding N_14_C-1373, and no induction time is required. This will be an outstanding clue for N_14_C-1373 use in real technologies applications.

As seen from Fig. 5c,d, both g-C_3_N_4_/lignin ratio and annealing temperature have significant effects on the reaction kinetics parameters of N_x_C-T catalysts, the apparent rate constants (*k*_*app*_) and specific mass activity. It is interesting to note that the N_14_C-1373 with the lowest total N content (3.3 atom %) shows the highest values of *k*_*app*_ (4.77 min^−1^) and specific mass activity (361 mol kg^−1^ h^−1^), probably due to the highest graphite N content (69.4%) and S_BET_ (1481.9 m^2^/g). It is speculated that graphite N species and S_BET_ of NC plays a critical role for impelling 4-NP reduction rather than the total N atoms content^19–21^. Apple-to-apple comparisons of catalytic activities of N_14_C-1173, N_14_C-1273 and N_14_C-1373, it is found that N_14_C-1173 (I_D_/I_G_ = 1.03) with the largest content of defective sites shows lower performance activity than N_14_C-1273 (I_D_/I_G_ = 1.00) and N_14_C-1373 (I_D_/I_G_ = 0.97), revealing that the catalytic activity of NC is not caused by the defect sites on the surface of catalysts, which is different from the results reported in the literature^31^. This contradictory phenomenon indicates that the effect of defect sites in NC on the catalytic performance needs further investigation in future.

**Figure 6:**
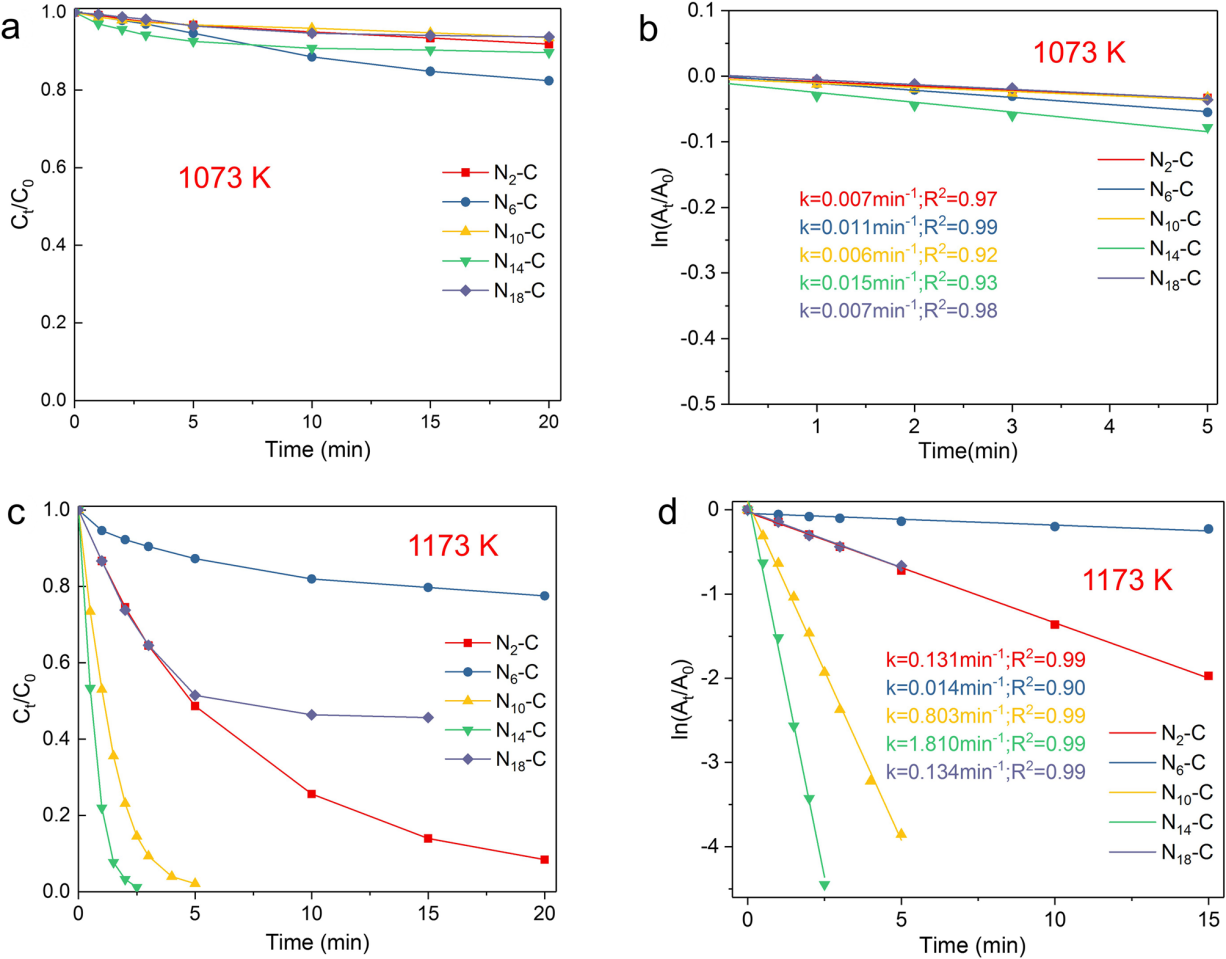

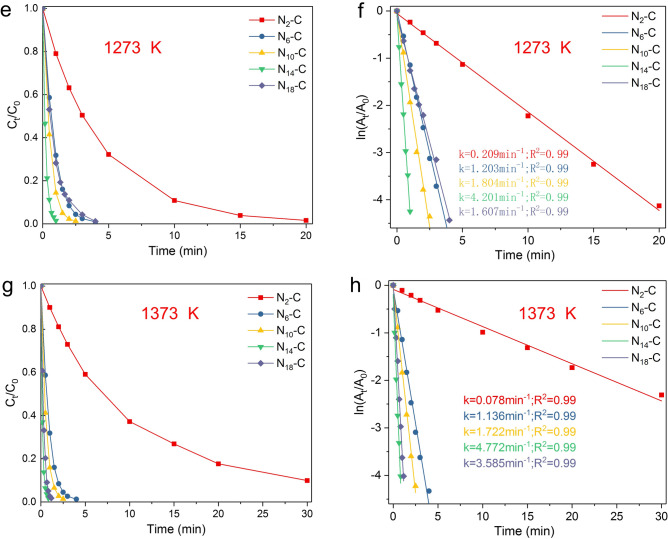
The reaction kinetics of the as-prepared NC catalysts at different g-C_3_N_4_/lignin ratio and annealing temperature for 4-NP reduction. The kinetic apparent rate constant (*k*_*app*_) was calculated by using pseudo-first order kinetic model (${ - }k_{app} t = \ln \frac{{C_{t} }}{{C_{0} }}$). The experimental data of *C*_*t*_*/C*_*0*_ versus *t* are shown in (**a**,**c**,**e**,**g**) (left). The fitting plot curves of ln (*C*_*t*_*/C*_*0*_ ) versus t are shown in (**b**,**d**,**f**,**h**) (right).

The reaction kinetics of twenty catalysts prepared at different g-C_3_N_4_/lignin ratio and annealing temperature were conducted for 4-NP reduction, the kinetics experimental data were expressed by $\frac{{C_{t} }}{{C_{0} }} \to t$ (Fig. 6a,c,e,g). The assumption of pseudo-first-order kinetics is supported by the data of the linear fit between ln [C_t_/C_0_] and reaction time (t) (Fig. 6b,d,f,h) in the presence of a large molar excess of NaBH_4_. The reaction following pseudo-first-order kinetics is different from N-doped graphene oxide^19,23^, while it is similar to metallic catalysts^22,24–26^. Typically, the catalytic reduction of 4-NP over metallic catalysts is ascribed to a pseudo-first-order reaction^22,24–26^. N-doped graphene leads to the pseudo-zero-order reaction due to the limited number of active sites^19,23^. As described afored, N-doping species rather than the total N content will influence the catalytic performance of NC catalysts^19,23^.

**Figure 7:**
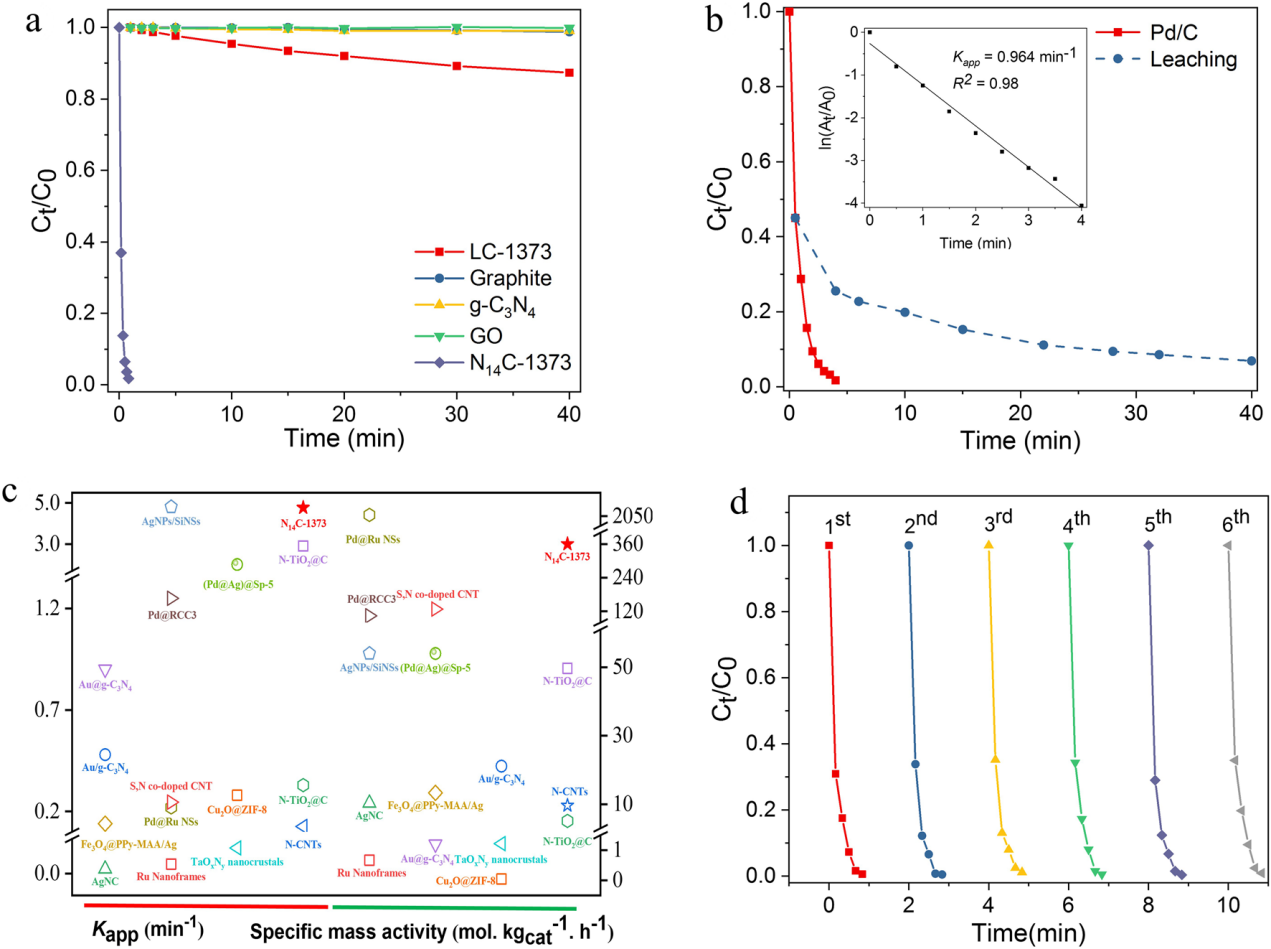
Activities comparison and stability of the as-prepared N_14_C-1373. (**a**) Performance comparison of several counterparts catalysts. (**b**) Catalytic performance of commercial Pb/C catalyst. (**c**) Kinetics parameters comparison of N_14_C-1373 with other catalysts reported in the literature. (**d**) Re-usability of N_14_C-1373 after 6 runs.

To further insight into the effect of nitrogen dopant on the activity of lignin-derived NC catalysts, several counterparts including LC-1373, graphite powder, g-C_3_N_4_, graphene oxide (GO), and N_14_C-1373 were compared for the conversion of 4-NP to 4-AP (Fig. 7a). In the absence of N-dopant, LC-1373, graphite and GO show little catalytic activity. It is also true for neat g-C_3_N_4_. These results strongly confirm that carbon atom without N-dopant and neat g-C_3_N_4_ have little contribute to 4-NP reduction reaction (Fig. 7a; Supplementary Table S3). That is to say, N-dopant plays a critical role in 4-NP reduction reaction over the as-synthesized NC catalyst^19,23^. NC as metal-free catalyst can avoid this problem and does not suffer from secondary contamination caused by metallic catalyst, even for commercial metallic Pb/C catalyst, due to metal leaching (Fig. 7b), From the linear relationshp of $\ln (\frac{{C_{t} }}{{C_{0} }}) \to t$ sinet of Fig. 7b, metallic Pb/C catalyst shows pseudo-first-order kinetics reaction with *K*_*app*_ value of 0.084 min^−1^, far lower than N_14_C-1373 (*k*_*app*_ = 4.77 min^−1^) in our work. As summarized in Fig. 7c and Supplementary Table S4, the as-prepared N_14_C-1373 shows the highest performance activity in the 4-NP reduction, and greatly outperforms most previously reported catalysts in the literature, regards of metallic and metal-free catalysts. Although N_14_C-1373 shows excellent activity, however, the operational stability of the catalyst is equally vital to its activity. Thus, we investigated the durability of N_14_C-1373 by recycling the catalyst and measuring its activity. Impressively, N_14_C-1373 is highly reusable after six cycles without any loss of activity (Fig. 7d), revealing its outstanding stability and potential practical application in the coming years.

### DFT calculation of 4-NP reduction over N_14_C-1373

**Figure 8:**
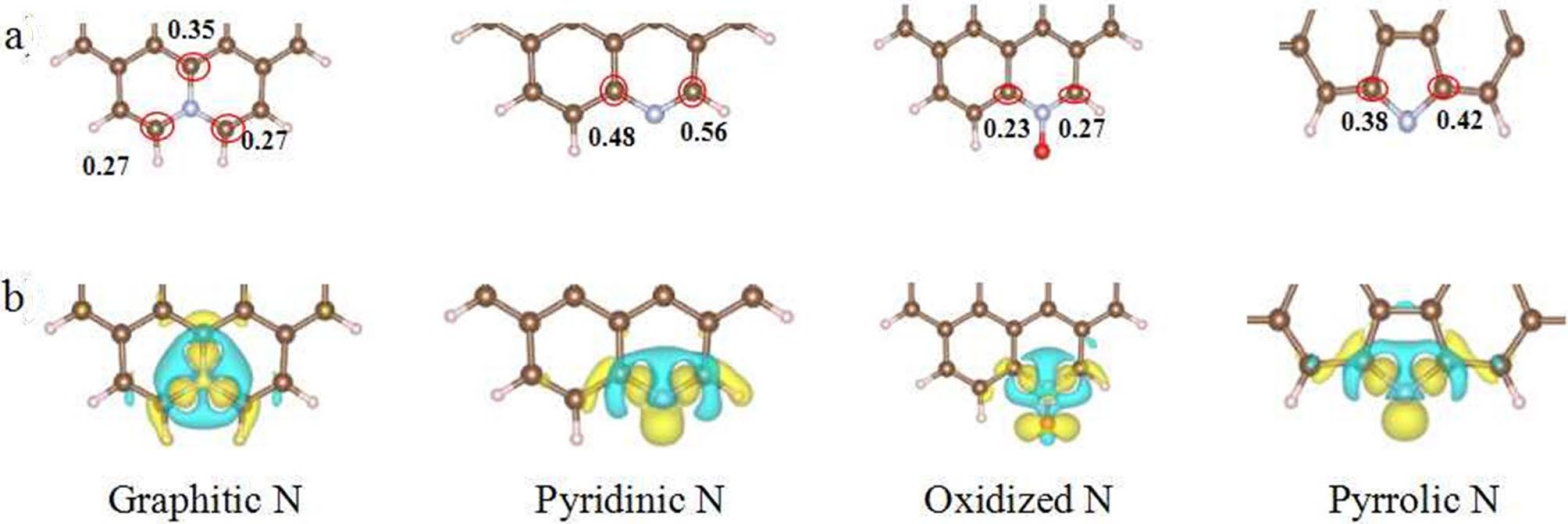
Bader charge and difference of charge of the as-prepared NC. (**a**) Bader charge, the digital number means the positive charge of carbon; (**b**) difference of charge. Yellow color represents charge accumulation, blue color indicates charge loss.

To address the catalytic mechanism of N_14_C-1373 for 4-NP reduction, it was investigated the effect nitrogen dopants on the electronic structure of N_14_C-1373 through DFT calculation. From the Bader charge and difference of charge in Fig. 8, the doped N atoms can introduce local high positive charge density and high spin density to their adjacent carbon atoms on the N_14_C-1373 surface, leading to the carbon atoms activation with positive charges, which confers the carbon a metal-like d band electronic structure, and thus, a metal-like catalytic performance^18^, its reaction kinetics can be described by the Langmuir isotherm^23^. Incorporation of the nitrogen dopants species will induce the charge redistribution and make the adjacent carbon bear a different positive charge (Fig. 8a). The greater of the number value in Fig. 8a is, the higher positive charge will be. For the difference of charge of graphitic, pyridinic and pyrrolic N in Fig. 8b, charge accumulates in N–C bonds. While for oxidized N species, apart form N–C bonds of charge accumulation, some charge is accumulating in oxygen atom.

**Figure 9:**
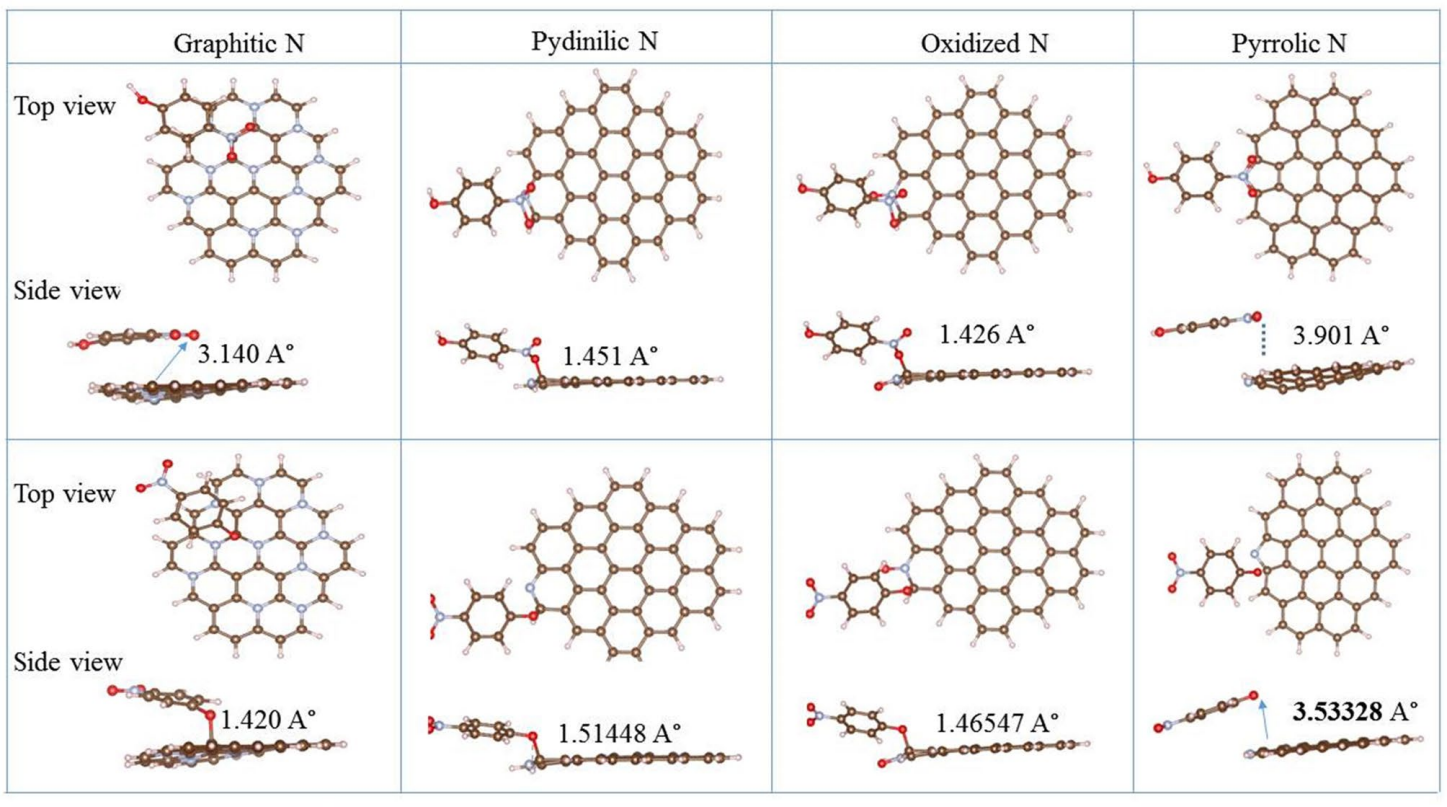
The optimized structures of 4-NP ions adsorbed on N_14_C-1373: on the top line is nitro-group binding with graphitic, pyridinic, oxidized and pyrrolic N species; and the bottom line stands O atom in hydroxyl group binding with (graphitic, pyridinic, oxidized and pyrrolic N species. The separated distances for each model have been marked directly in the corresponding figures.

Subsequently, the precise adsorption model of 4-NP over N_14_C-1373 was proposed, and the contribution of the doped N species to catalytic performance was also evaluated using DFT. The optimized N_14_C-1373 model is shown in Supplementary Fig. S3. Since the carbon active site on N_14_C-1373 is positively charged due to the large electron negativity of the doped N atoms, the O atom of 4-NP is the preferred binding site^19,23^. Because 4-NP ion has two binding sites, nitro group and the O atom of hydroxyl substituents, it is further investigated the adsorption bond length and free energies of 4-NP ion over N_14_C-1373 via nitro group and the O atom of hydroxyl substitutes, respectively. From the adsorption energies data in Supplementary Table S5, regardless of nitro group and the O atom of hydroxyl substituents binding sites, 4-NP adsorption reaction is spontaneous due to the negative adsorption energies. It indicates the 4-NP reduction reaction is too fast to detect the intermediate products. This explains the fact of the complete conversion of 4-NP to 4-AP without the generation of byproducts (Fig. 5b). From the view point of bond length and adsorption energies (Fig. 9; Supplementary Table S5), a meaningful conclusion has been drawn that four kinds of N dopants can improve the 4-NP adsorption ability of N_14_C-1373 with different contribute to the catalytic properties. If the O atom of hydroxyl subtituents is the binding site, the graphitic N dopant species show the highest significant contribution to the catalytic activity due to its lowest adsorption free energy (△E = − 3.6470) and shortest adsorption bonding length (1.462 Å). On the contrary, considering nitro group as binding site, the oxidized N species exhibits the highest significant contribution to the catalytic activity due to its lowest adsorption free energy (△E = − 0.8261) and shortest adsorption bonding length (1.426 Å).

**Figure 10:**
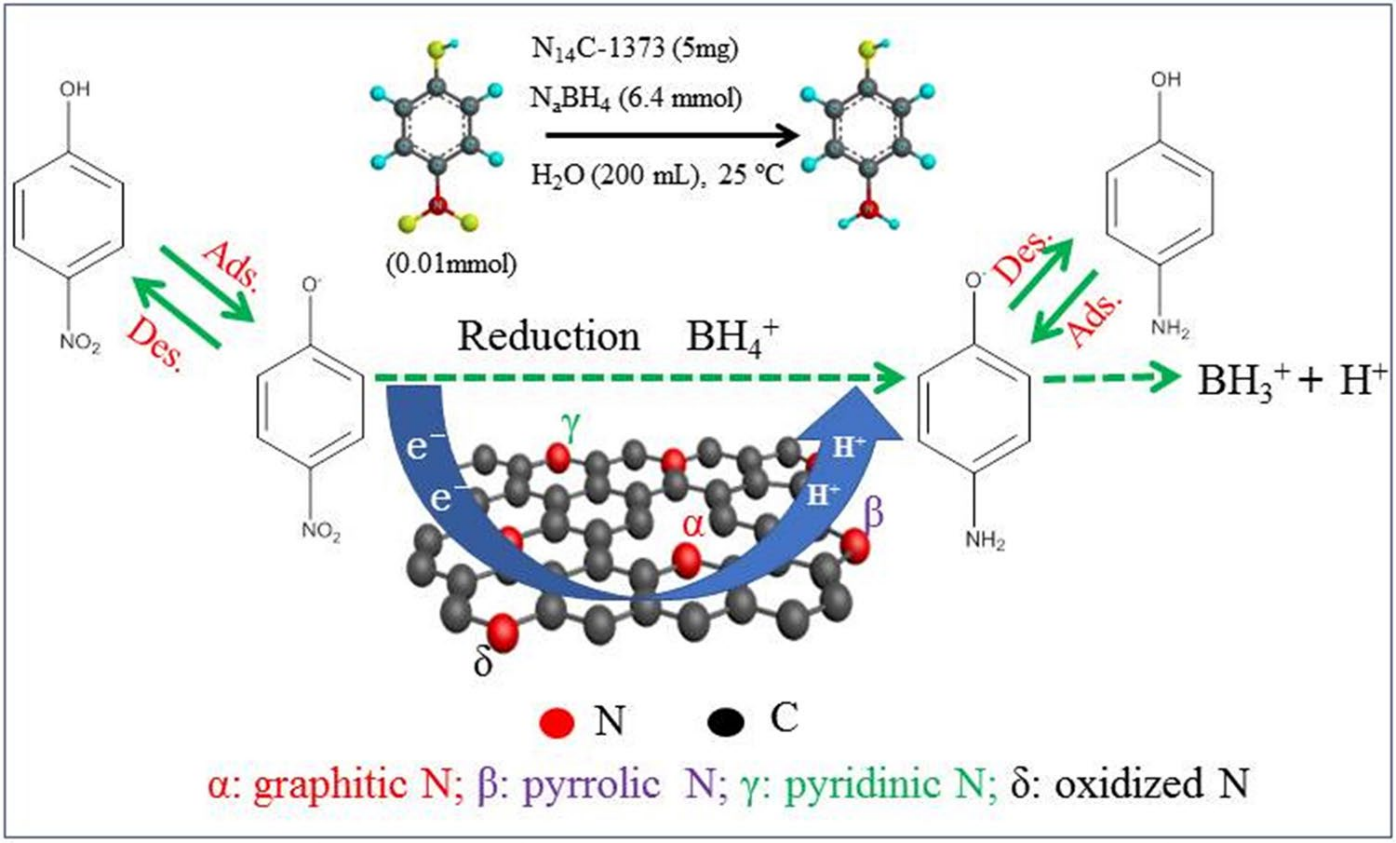
Possible catalytic mechanism of 4-NP reduction over lignin-derived N_14_C-1373 using NaBH_4_ as hydrogen donor in water at room temperature.

Basd on experimental data and DFT calculation, a plausible catalytic mechanism for 4-NP reduction over N_14_C-1373 has been proposed and shown in Fig. 10. In the first step, hydrogen atom releasing from the adsorbed BH_4_ was adsorbed on the surface of N_14_C-137, simultaneously, 4-nitrophenolate ions are also absorbed on the carbon atoms active sites via nitro groups, which are activated for subsequent reduction. In the second step, 4-nitrophenolate ions are reduced by surface hydrogen species into the 4-aminophenolate ions intermediates. Then, 4-aminophenolate ions are absorbed on the carbon atoms active sites via the O atom of hydroxyl substituents. In the fourth step, 4-aminophenolate ions are reduced by surface hydrogen species into neutral 4-AP, which will desorption from the surface of N_14_C-1373 surface and it creates a free surface for the next catalytic cycle. Therefore, the adsorption of 4-nitrophenolate ions is very critical for 4-NP reduction over N_14_C-1373. A similar mechanism of 4-NP reduction has been found for metallic catalysts and N-doped graphite in the literature^22–26^. It is worthily noticed that the as-prepared N_14_C-1373 has metallic properties with ample active sites, and the rates of substrate adsorption and product desorption are quite fast. Thus, the reduction kinetics is of pseudo-first-kinetics reaction, which can be described by the Langmuir isotherm^20,21^. In addition, the defect-rich structure of N_14_C-1373 surface is not helpful to electronic transferring^42^, which leads to low activity of catalyst. In contrast, the high BET surface area of catalyst results in strong adsorption ability and high-efficient electronic transferring, which can improve catalytic activity. In addition, N_14_C-1373 with high concentration (69 at%) of graphitic N exhibits high electronic transfer activity for hydrogenation reaction^43^. These phenomena are in good agreement with the experimental data in Fig. 5c,d.

## Conclusions

In summary, lignin-derived NC as metal-free catalyst has been successfully synthesized using lignin as carbon source and g-C_3_N_4_ as sacrificial template under different g-C_3_N_4_/lignin ratio and annealing temperature conditions. These NCcatalysts are evaluated for the catalytic reduction of 4-NP to 4-AP via hydrogen transfer in NaBH_4_ aqueous system. Characterization detailed information shows that the as-prepared N_14_C-1373 has the highest S_BET_ (1481.9 m^2^/g) and graphitic nitrogen (69 at%). Kinetics investigation of 4-NP reduction over N_14_C-1373 shows that it follows first-order reaction kinetics with *k*_app_ = 4.77 min^−1^ and specific mass activity (s = 361 mol. kg_cat_^−1^ h^−1^), which are the best values to date for 4-NP reduction. 4-NP ions adsorption has an essential influence on catalytic reduction, which initials spontaneously in the presence of N_14_C-1373 catalyst. The charge density and adsorption models from DFT calculations demonstrate that the active sites of N_14_C-1373 for 4-NP reduction are the carbon atoms adjacent to the N dopants, which contribute to catalytic performance in dependence of N dopants species, among which graphitic N species has much greater contribution to 4-NP adsorption. Because the nitrogen dopants can change the electronic structure of the adjacent carbon atoms and promote the chemical activity, DFT calculations verify that 4-NP ions will combine with active sites on N_14_C-1373 surface via both nitro group and the O atom of hydroxyl group on the condition of low graphitic N species content. Interestingly, the as-prepared N_14_C-1373 has excellent stability and good re-usability, as well as minimal contamination issues in comparison with commercial Pb/C catalyst.

## Supplementary information

Supplementary Information
