# Supplementary material for: Synthesis of lignin-derived nitrogen-doped carbon as a novel catalyst for 4-NP reduction evaluation
Source: Sci Rep. 2020 Nov 18;10:20075. doi: 10.1038/s41598-020-76039-9 (PMC7675980; doi:10.1038/s41598-020-76039-9)
Supplement: Supplementary file 1 — Supplementary Information [file 41598_2020_76039_MOESM1_ESM.doc]

**Supplementary data**

**Synthesis of lignin-derived nitrogen-doped carbon as a novel catalyst for 4-NP reduction evaluation**

Yun Liu^1,2,3,4*^, Huanghui Xu^1^, Hongfei Yu^1^, Haihua Yang^1^, Tao Chen ^2,3,4^

(^1^College of Life Science and Technology, Beijing University of Chemical Technology, Beijing 100029, China;

^2^ School of Nuclear Technology and Chemistry & Biology, Hubei University of Science and Technology, Xianning 437100, China;

^3^Hubei Key Laboratory of Radiation Chemistry and Functional Materials, Hubei University of Science and Technology, Xianning 437100, China;

^4^ Hubei Engineering Research Center for Fragrant Plants, Hubei University of Science and Technology, Xianning 437100, China)


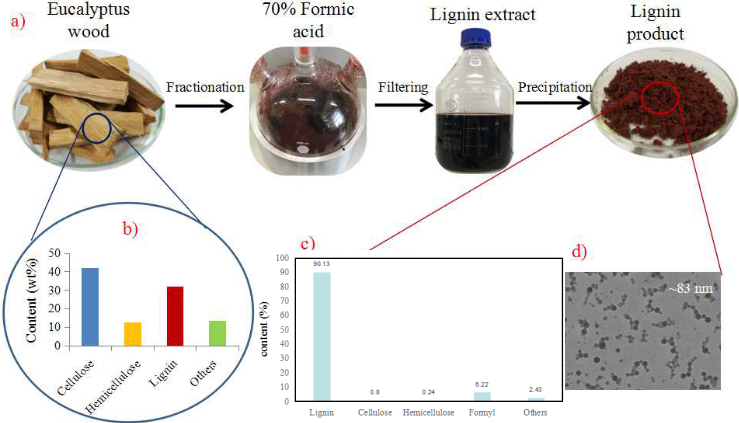


**Fig.S1** Lignin isolated from biomass through formic acid fractionation and its compositions. (a) The procedure of lignin fractionation from eucalyptus wood using 70% formic acid aqueous solution; (b) Compositions of Eucalyptus wood; (c) Compositions of lignin product; (d) Morphology observation of lignin product by transmittance electronic microscope (TEM).


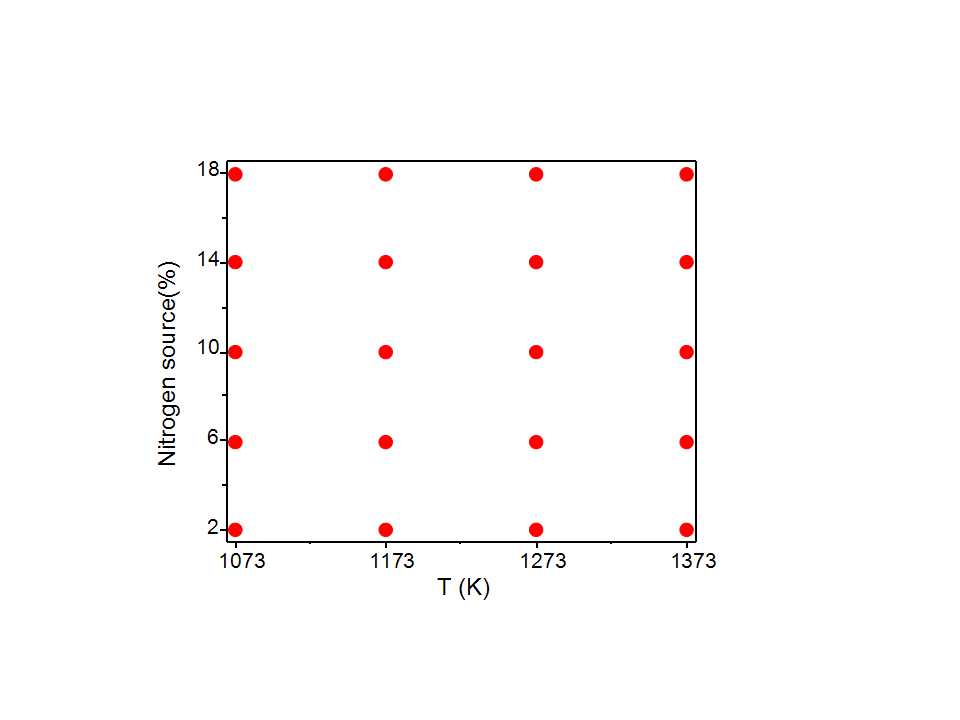


**Fig. S2** Experimental design of N_x_C-T catalysts syntheses as the function of annealing temperature (T) and nitrogen amount (N%, g-C_3_N_4_/lignin mass ratio)


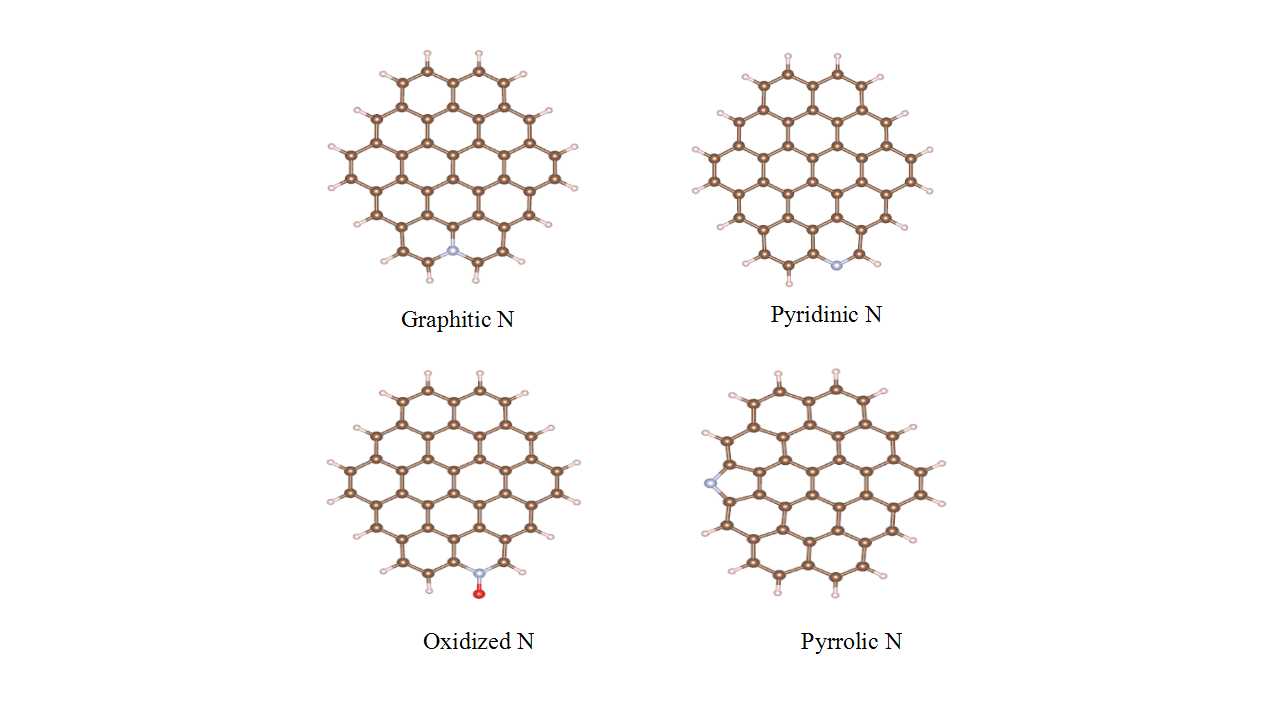


**Fig. S3** The optimized N_14_C-1373 model through N-doped graphene cluster models (20 Å × 20 Å ×12 Å)


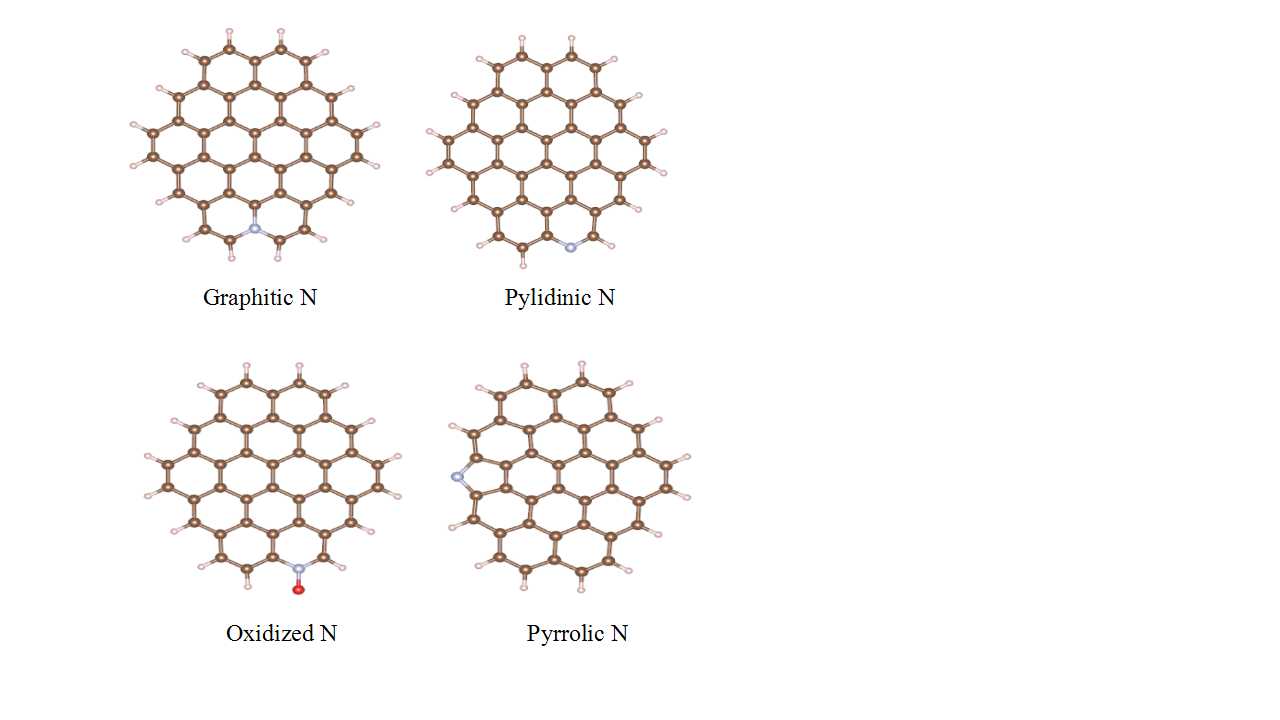


**Table S1** Twenty kinds of N_x_C-T catalysts as the function of annealing temperature (T) and nitrogen content (N%, g-C_3_N_4_/lignin mass ratio)

| No. | Catalyst name | Annealing temperature (K) | Nitrogen content (%) |
| --- | --- | --- | --- |
| 1 | N_2_C-1073 | 1073 | 2 |
| 2 | N_6_C-1073 | 1073 | 6 |
| 3 | N_10_C-1073 | 1073 | 10 |
| 4 | N_14_C-1073 | 1073 | 14 |
| 5 | N_18_C-1073 | 1073 | 18 |
| 6 | N_2_C-1173 | 1173 | 2 |
| 7 | N_6_C-1173 | 1173 | 6 |
| 8 | N_10_C-1173 | 1173 | 10 |
| 9 | N_14_C-1173 | 1173 | 14 |
| 10 | N_18_C-1173 | 1173 | 18 |
| 11 | N_2_C-1273 | 1273 | 2 |
| 12 | N_6_C-1273 | 1273 | 6 |
| 13 | N_10_C-1273 | 1273 | 10 |
| 14 | N_14_C-1273 | 1273 | 14 |
| 15 | N_18_C-1273 | 1273 | 18 |
| 16 | N_2_C-1373 | 1373 | 2 |
| 17 | N_6_C-1373 | 1373 | 6 |
| 18 | N_10_C-1373 | 1373 | 10 |
| 19 | N_14_C-1373 | 1373 | 14 |
| 20 | N_18_C-1373 | 1373 | 18 |

**Table S2** Element analysis and N species of N_x_C-T catalysts based on XPS spectra

| Catalysts | Element content (atom %) | | | N species content (atom %) | | | |
| --- | --- | --- | --- | --- | --- | --- | --- |
|  | N | O | C | Graphitic N | Pyridinic N | Pyrrolic N | Oxidized N |
| N_14_C-1073 | 14.80 | 3.53 | 81.67 | 3.87 | 6.63 | 2.97 | 1.33 |
| N_14_C-1173 | 8.04 | 7.08 | 84.88 | 3.59 | 2.88 | 1.19 | 0.38 |
| N_14_C-1273 | 4.84 | 5.71 | 89.44 | 2.53 | 1.46 | 0.57 | 0.28 |
| N_14_C-1373 | 3.30 | 5.37 | 91.33 | 2.29 | 0.82 | 0.10 | 0.10 |
| LC-1373 | 0.58 | 8.28 | 91.14 | n.a | n.a | n.a | n.a |
| N_2_C-1373 | 3.48 | 2.45 | 94.07 | 2.29 | 0.82 | 0.10 | 0.10 |
| N_6_C-1373 | 3.45 | 5.39 | 91.16 | 2.29 | 0.82 | 0.10 | 0.10 |
| N_10_C-1373 | 3.11 | 4.57 | 92.32 | 2.29 | 0.82 | 0.10 | 0.10 |
| N_14_C-1373 | 3.30 | 5.37 | 91.33 | 2.29 | 0.82 | 0.10 | 0.10 |
| N_18_C-1373 | 2.88 | 6.31 | 90.81 | 2.29 | 0.82 | 0.10 | 0.10 |

**Table S3**  Catalytic performance of different carbon-based metal-free catalysts for 4-NP reduction

| Catalyst | Time  (min) | C_t_/C_0_ | *k*_app_  (min^-1^) | Sspecific mass activity  (mol.kgcat^-1^.h^-1^) |
| --- | --- | --- | --- | --- |
| No catalyst | 40 | 1 | / | / |
| LC-1373 | 40 | 0.87 | 0.005 | 0.836 |
| g-C_3_N_4_ | 40 | 1 | / | / |
| Graphite | 40 | 1 | / | / |
| GO | 40 | 1 | / | / |
| N_14_C-1373 | 0.67 | 0.05 | 4.77 | 361 |

**Table S4** The performance of catalyst for 4-NP reduction with literatures

| Catalysts | T  (℃) | *k_app_*  (min^-1^) | Conversion /time (%/min) | Specific mass activity (mol.kg_cat_^-1^.h^-1^) | | | Refs. |
| --- | --- | --- | --- | --- | --- | --- | --- |
| Metallic catalysts | | | | | | | |
| Ru Nanoframes | 25 | 0.022 | 74/60 | 0.67 | | [1] | |
| Au/g-C_3_N_4_ |  | 0.48 | 98/10 | 21.1 | | [2] | |
| AgNC | 25 | 0.01077 | 100/120 | 10.33 | | [3] | |
| Au@g-C_3_N_4_ |  | 0.9 | 100/10 | 1.2 | | [4] | |
| Fe_3_O_4_@PPy-MAA/Ag | 25 | 0.14 | 100/45 | 13.33 | | [5] | |
| TaO_x_N_y_ nanocrystals | 25 | 0.059 | 91.3/45 | 1.22 | | [6] | |
| Pd@RCC3 | 25 | 1.25 | 100/4 | 104 | | [7] | |
| Pd@Ru NSs | RT | 0.22 | 90/14 | 2053 | |  | |
| Cu_2_O@ZIF-8 | 25 | 0.28 | 99/14 | 0.0442 | | [9] | |
| AgNPs/SiNSs | RT | 4.81 | 100/0.67 | 54 | | [10] | |
| (Pd@Ag)@Sp-5 | RT | 2.01 | 100/4 | 5.4 | | [11] | |
| CuO-ZnO-ESM | RT | 0.7919 | 98/12 | / | | [12] | |
| Metal-free catalysts | | | | | | | |
| N_14_C-1373 | RT | 4.77 | 95/0.67 | 361 | This work | | |
| S,N co-doped CNT | 20 | 0.246 | 100/10 | 128 | [13] | | |
| N-CNTs | RT | 0.126 | 100/18.7 | 9.62 | [14] | | |
| N-TiO_2_@C | 25 | 0.33 | 99/10 | 5.13 | [15] | | |
|  | 55 | 2.91 | 96/1 | 49.7 |  |  |  |

**Table S5** Adsorption energies and bonding length of 4-NP over N14C-1373 via nitro group as binding site

| Nitro group as binding site | Pyridinic N | Oxidized N | Pyrrolinic N | Graphitic N |
| --- | --- | --- | --- | --- |
| E_base_ | -436.9295 | -442.2964 | -422.03235 | -421.8760 |
| E_intermediate_ | -95.8624 | -95.8624 | -95.8624 | -95.8624 |
| E_total_ | -533.1989 | -538.9849 | -520.3538 | -518.1520 |
| ΔE | -0.4069 | -0.8261 | -2.4590* | -0.4130 |
| Bonding length(Å) | 1.451 | 1.426 | 3.901 | 3.140 |

Note: Δ*E* = *E_total_* –E_Ebase_-E_intermediate_.

* low Δ*E value* maybe caused by structural change

**Table S6** Adsorption energies and bonding length of 4-NP over N14C-1373 via the O atom of hydroxyl group as binding site

| O atom of hydroxyl group as binding site | Pyridinic N | Oxidized N | Pyrrolinic N | Graphitic N |
| --- | --- | --- | --- | --- |
| E_base_ | -436.9295 | -442.2964 | -422.0323 | -421.8760 |
| E _intermediate_ | -90.6561 | -90.6561 | -90.6561 | -90.6561 |
| E_total_ | -530.0293 | -535.9023 | -515.2553 | -516.1800 |
| ΔE | -2.4437 | -2.9499 | -2.5669 | -3.6470 |
| Bonding length(Å) | 1.5145 | 1.4655 | 3.5333 | 1.4620 |

Note: Δ*E* = *E_total_* –E_Ebase_-E_intermediate_.

**References**

[1] Ye, H., Wang, Q., Catalano, M., Lu, N., Vermeylen, J., Kim, M.J., Liu, Y., Sun, Y., & Xia, X. Ru Nanoframes with an fcc Structure and Enhanced Catalytic Properties. *Nano Lett*. **16**, 2812-2817(2016).

[2] Fu, Y., Huang, T., Jia, B., Zhu, J., Wang, X. Reduction of nitrophenols to aminophenols under concerted catalysis by Au/g-C_3_N_4_ contact system. *Appl. Catal. B-Environ*. **202,** 430-437(2017).

[3] Giri, S., Das, R., van der Westhuyzen, C., Maity, A. An efficient selective reduction of nitroarenes catalyzed by reusable silver-adsorbed waste nanocomposite. *Appl. Catal. B-Environ.* **209**, 669-678(2017).

[4] Nguyena, T., Huang, C., Doong, R.A. Enhanced catalytic reduction of nitrophenols by sodium borohydride over highly recyclable Au@graphitic carbon nitride nanocomposites. *Appl. Catal. B-Environ*. 240, 337-347(2019).

[5] Das, R., Sypu, V.S., Paumo, H.K., Bhaumik, M., Maharaj, V., Maity, A. Silver decorated magnetic nanocomposite (Fe_3_O_4_@PPy-MAA/Ag) as highly active catalyst towards reduction of 4-nitrophenol and toxic organic dyes. *Appl. Catal. B- Environ*. **24**, 546-558(2018) .

[6] Su, Y., Lang, J., Li, L., Guan, K., Du, C., Peng, L., Han, D., & Wang, X. Unexpected catalytic performance in silent tantalum oxide through nitridation and defect chemistry. *J. Am. Chem. Soc.* 135, 11433-11436(2013) .

[7] Yang, X., Sun, J.K., Kitta, M., Pang, H., & Xu, Q. Encapsulating highly catalytically active metal nanoclusters inside porous organic cages. *Nat. Catal.* **1**, 214-220(2018).

[8] Zhang, Z., Liu, Y., Chen, B., Gong, Y., Gu, L., Fan, Z., et al Submonolayered Ru Deposited on Ultrathin Pd Nanosheets used for Enhanced Catalytic Applications. *Adv. Mater.* **28**, 10282-10286(2016).

[9] Li, B., Ma, J.G., & Cheng, P. Silica-Protection-Assisted Encapsulation of Cu2 O Nanocubes into a Metal-Organic Framework (ZIF-8) To Provide a Composite Catalyst. *Angew. Chem. Int. Ed. Engl.* **57**, 6834-6837(2018).

[10] Yan, Z., Hu, L., Zuo, X., Yang, H. Green assembly of stable and uniform silver nanoparticles on 2D silica nanosheets for catalytic reduction of 4-nitrophenol. *Appl. Catal. B-Environ.* **226**, 23-30 (2018). https://doi.org/10.1016/j.apcatb.2017.12. 040.

[11] Sun, L., Zhang, D., Sun, Y., Wang, S., Cai, J. Facile Fabrication of Highly Dispersed Pd@Ag Core–Shell Nanoparticles Embedded in Spirulina platensis by Electroless Deposition and Their Catalytic Properties. *Adv. Func. Mat.* **28**, 1707231(2018).

[12] He, X., Yang, D.P., Zhang, X., Liu, M., Kang, Z., Lin, H., Jia, N., Luque, R. Waste Eggshell Membrane-Templated CuO-ZnO Nanocomposites with Enhanced Adsorption, Catalysis and Antibacterial Properties for Water Purification. *Chem. Eng. J*. **369**, 621-633(2019).

[13] Wang, F., Song, S., Li, K., Li, J., Pan, J., Yao, S., Ge, X., Feng, J., Wang, X., & Zhang, H. A "Solid Dual-Ions-Transformation" Route to S,N Co-Doped Carbon Nanotubes as Highly Efficient "Metal-Free" Catalysts for Organic Reactions. *Adv. Mater.* **28**, 10679-10683(2016).

[14] Gao, L., Li, R., Sui, X., Li, R., Chen, C., & Chen, Q. Conversion of chicken feather waste to N-doped carbon nanotubes for the catalytic reduction of 4-nitrophenol. *Environ. Sci. Technol*. **48**. 10191-10197(2014).

[15] Pan, X., Gao, X., Chen, X., Lee, H.N., Liu, Y., Withers, R.L., & Yi, Z. Design Synthesis of Nitrogen-Doped TiO2@Carbon Nanosheets toward Selective Nitroaromatics Reduction under Mild Conditions. *ACS Catal.* **7**, 6991-6998(2017).
